# Intravoxel incoherent motion diffusion-weighted MR imaging of the liver using respiratory-cardiac double triggering

**DOI:** 10.18632/oncotarget.21824

**Published:** 2017-10-11

**Authors:** Jinning Li, Caiyuan Zhang, Yanfen Cui, Huanhuan Liu, Weibo Chen, Guilong Wang, Dengbin Wang

**Affiliations:** ^1^ Department of Radiology, Xinhua Hospital, Shanghai Jiao Tong University School of Medicine, Shanghai 200092, China; ^2^ Philips Healthcare, Shanghai 200233, China

**Keywords:** intravoxel incoherent motion, diffusion-weighted imaging, liver, repeatability, respiratory-cardiac double triggering

## Abstract

To investigate the influence of respiratory-cardiac double triggering (RCT) on intravoxel incoherent motion (IVIM) diffusion-weighted imaging (DWI) for the liver, twelve healthy volunteers underwent liver DWI twice respectively with respiratory triggering (RT) and RCT schemes. Signal-to-noise ratios (SNRs) of the images, values, repeatability (evaluating with within-subject coefficient of variation), and variability of quantitative parameters, including apparent diffusion coefficient (ADC), pure diffusion coefficient (*D*), perfusion fraction (*f*), and perfusion-related diffusion coefficient (D*), were evaluated for each DWI sequence. Results showed that the use of RCT scheme significantly enhanced SNRs (*P* < 0.001), improved the measurement precision (*P* ≤ 0.023) and repeatability (*P* ≤ 0.009) of ADC, *D*, and *f* values, decreased the variability of ADC and D values (*P* ≤ 0.015). Furthermore, this improvement was not completely confined to the left liver lobe, but also observed for the right liver lobe. Moreover, the precision of D* values in the right lobe (*P* < 0.001) and its repeatability in the left lobe (*P =* 0.002) were also significantly improved. Thus, our findings suggest that RCT is a more effective physiological scheme for improving SNRs, the precision, repeatability, and variability of quantitative parameters than RT for IVIM-DWI in the liver.

## INTRODUCTION

As a rapidly developing functional sequence, diffusion-weighted (DW) magnetic resonance (MR) imaging could not only provide the overall diffusion and microperfusion information in biological tissues, the apparent diffusion coefficient (ADC), but also separated pure diffusion coefficient (*D*), perfusion fraction (*f*), and perfusion-related diffusion coefficient (D*), which based on the theory of intravoxel incoherent motion (IVIM) [1, 2]. These quantitative parameters have been increasingly reported as promising tools for diagnostic work-up of the cranial and extracranial diseases [3–6]. Meanwhile, however, the issue of lower signal-to-noise ratios (SNRs) and poorer repeatability of ADC and IVIM parameters have also been pointed out to limit the extensive use of the quantitative analysis, especially for the liver [7, 8].

It is well known that the MR imaging of the liver is susceptive to physiology motions, such as respiratory and cardiac motion, which leads to signal loss, motion artifacts, blurring images, and the variability of quantitative parameters. As respiratory motion is the most common impact factor among all physiological motions, breath-holding or respiratory triggering (RT) has been generally applied to DW imaging (DWI) [9, 10]. However, the necessity to employ additional cardiac triggering has not reached a consensus.

On the one hand, several studies have depicted the less precision and poorer repeatability of ADC and IVIM parameters in the left liver lobe, caused by the propagation of cardiac motion to the liver [11, 12]. And single cardiac triggering has been shown the potential of decreasing the heart-related over-estimation and regional variability of ADC, *D*, and *f* values for the left liver lobe, but failed to significantly improve the test-retest repeatability of ADC and IVIM parameters [13]. On the other hand, it has also been concerned that the additional cardiac triggering would inevitably prolong the acquisition time and increase the risk of patient movements, which may offset the benefit of using double triggering scheme [13, 14]. Hence, we need to know what’s the potential benefit of combined respiratory and cardiac triggering scheme and whether it is required for clinical IVIM-DWI acquisition of the liver.

Therefore, in this prospective study, we aim to explore the influence of respiratory-cardiac double triggering (RCT) on image SNRs, the values of ADC and IVIM parameters and their repeatability and variability in the liver compared with RT scheme.

## RESULTS

### Acquisition time

The range of acquisition time was 6–9 min for RT DWI, and was 6–11 min for RCT DWI. The prolonged time for the additional cardiac triggering varied according to the respiratory and cardiac rate of each subject and was 1.3 min (range, 0–4 min) for the subjects in this study. For most of the respiratory cycle, the expiration time was long enough for triggering the signal acquisition in RCT DWI.

### SNR

**Figure 1:**
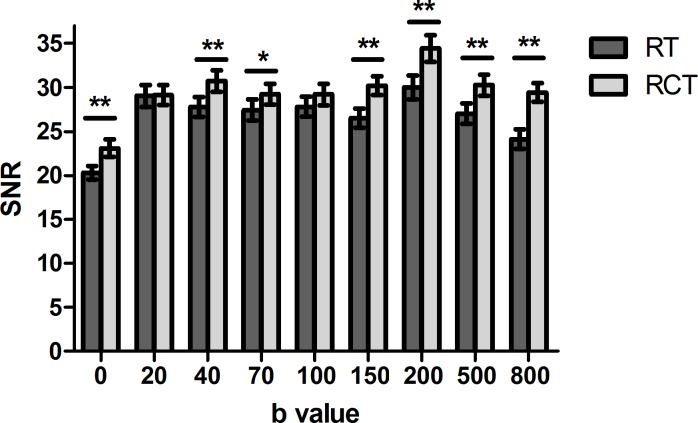
Averaged SNRs from two sessions of DW images acquired with RT and RCT scheme for each *b* value *P < 0.05, **P < 0.01.

For RT DWI, the SNR was 16.71–24.75 in the left liver lobe and 22.69–33.49 in the right liver lobe. For RCT DWI, the SNR was respectively 19.76–30.41 and 25.7–34.1 in the left and right liver lobe. Significant difference of SNR was detected between the two lobes for both two methods at each *b* value (*P* < 0.001). Figure 1 showed SNRs of images acquired at each *b* value by each scheme, and revealed that SNRs decreased with the increase of *b* value, especially for RT DWI with *b* ≥ 500 s/mm^2^ in this study. The use of RCT DWI significantly improved the SNRs of images (*P* < 0.001), except for the images with *b* value of 20 s/mm^2^ (*P* = 0.874) and of 100 s/mm^2^ (*P* = 0.057). Furthermore, the improvement tended to be more obvious for the left liver lobe and for the images with relatively high *b* values (*b* ≥ 150 s/mm^2^). The sudden increase of SNRs at *b* = 200 s/mm^2^ may be due to the high *b* values averaged twice (*b* ≥ 200 s/mm^2^).

### ADC and IVIM parameters

**Figure 2:**
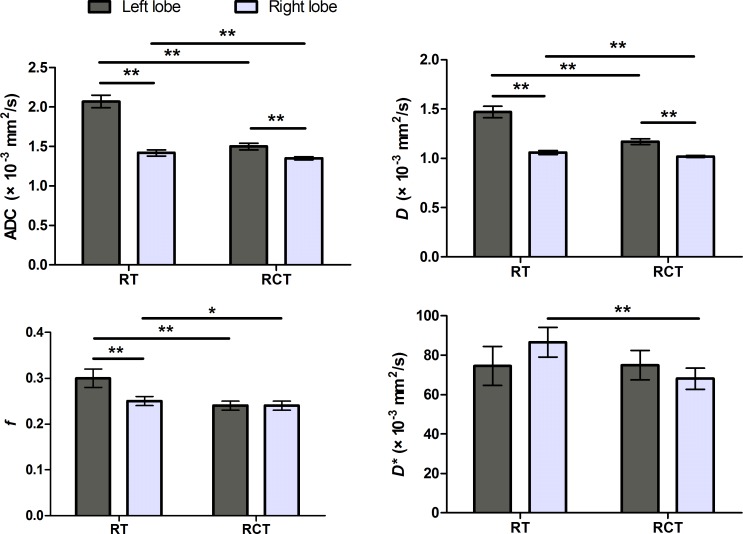
ADC and IVIM parameters measured at the left and right liver lobe with two DWI schemes There was a trend towards significantly higher values measured in the left liver lobe than those in the right, except *f* values in RCT scheme and D* values for both schemes. The difference of the parametric values between the two lobes was decreased for RCT DWI. *P < 0.05, **P < 0.01.

As shown in Table 1, there is significant difference of ADC and IVIM parameters of the liver between two DWI schemes. For both sessions, RCT DWI tended to result in significantly lower ADC and IVIM parameters than RT DWI (*P* < 0.001), except D* values in session 2 (*P* = 0.559). For the left liver lobe, the ADC, *D*, and *f* values had significant difference between two schemes (*P* < 0.001, Figure 2). Although the difference of ADC, *D*, and *f* values between two schemes was less obvious for the right liver lobe, which was shown in Figure 2, statistical differences were also detected for all parametric values (*P* ≤ 0.023).

**Table 1:** ADC and IVIM parameters calculated for two DWI sequences

Sequences	ADC (×10^−3^ mm^2^/s)	*D* (×10^−3^ mm^2^/s)	*f*	D* (×10^−3^ mm^2^/s)
**First Session**				
RT	1.68 ± 0.5	1.22 ± 0.34	0.27 ± 0.1	83.87 ± 60.45
RCT	1.42 ± 0.23	1.08 ± 0.15	0.25 ± 0.08	64.64 ± 37.2
*P*	< 0.001	< 0.001	< 0.001	< 0.001
**Second Session**				
RT	1.68 ± 0.5	1.23 ± 0.33	0.27 ± 0.1	79.78 ± 55.57
RCT	1.39 ± 0.19	1.09 ± 0.14	0.23 ± 0.07	77.05 ± 46.17
*P*	< 0.001	< 0.001	< 0.001	0.559

Data are mean ± standard deviations.

### Repeatability

**Figure 3:**
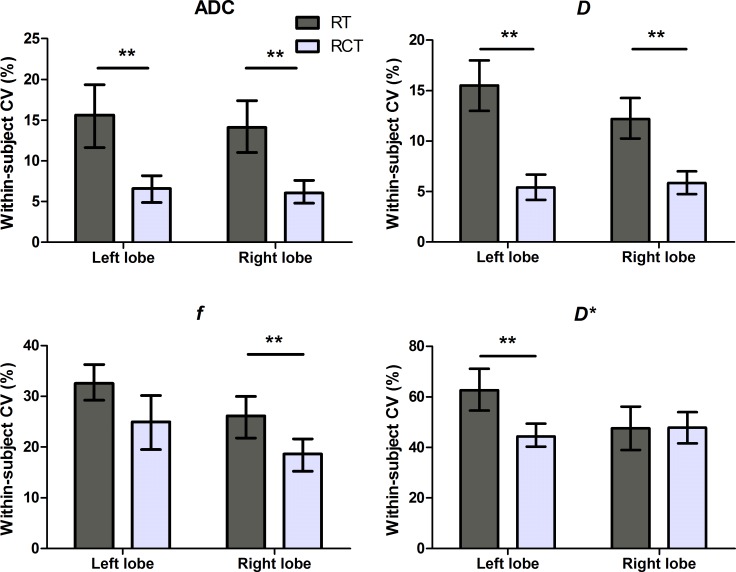
Repeatability of ADC and IVIM parameters with RT and RCT scheme for each liver lobe Note RCT DWI tended to result in improved repeatability of ADC and IVIM parameters, which is significant for ADC and *D* values of both liver lobes, *f* values of the right liver lobe, and for D* values of the left liver lobe. **P < 0.01.

The measurements of all the parametric values tend to result in lower within-subject coefficient of variation (CV) in the right liver lobe than those in the left liver lobe (Figure 3). Among ADC and IVIM parameters, the measurements of *D* (within-subject CV, 5.4%–15.51%) and ADC values (within-subject CV, 6.07%–15.62%) had a strong agreement between the two time points, while D* showed the poorest repeatability (within-subject CV, 44.27%–62.7%). For both liver lobes, compared with RT scheme, the use of RCT scheme tended to significantly improve the repeatability for ADC and IVIM parameters (*P* ≤ 0.009), except for *f* values of the left lobe (*P* = 0.06) and D* values of the right lobe (*P* = 0.956).

### Variability

**Figure 4:**
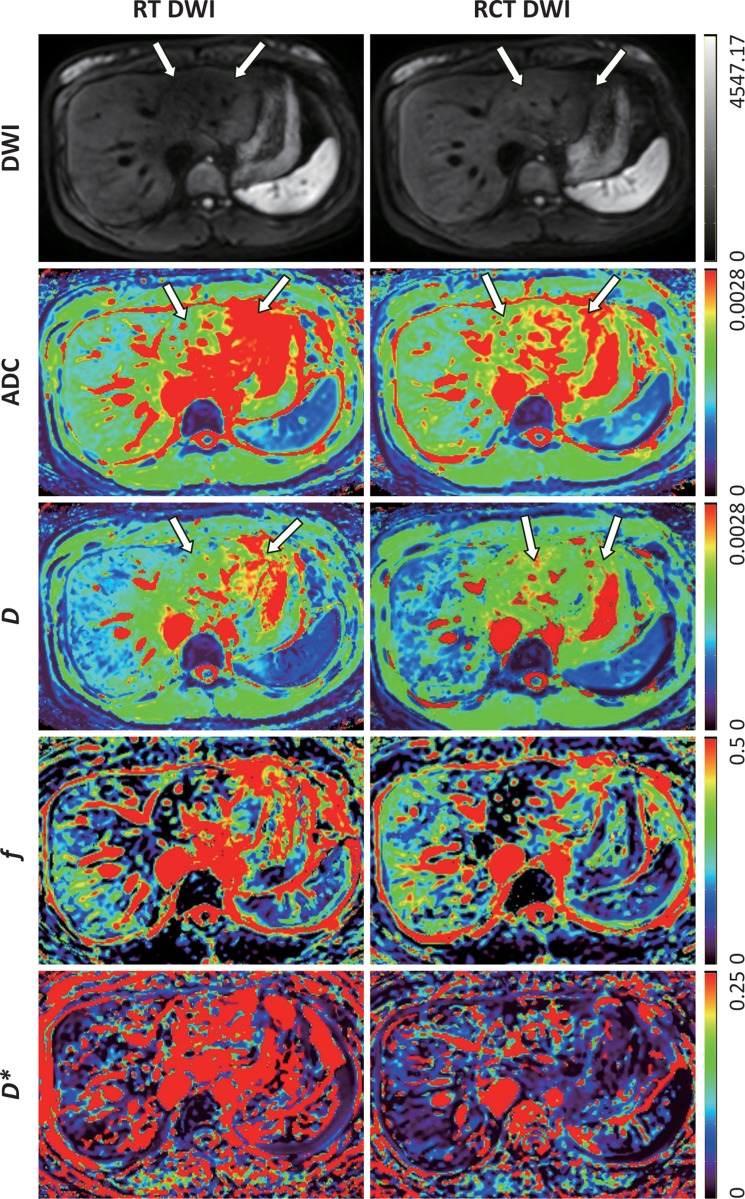
Axial diffusion-weighted MR images (*b* = 500 s/mm^2^) and ADC and IVIM parametric maps obtained by using RT and RCT scheme in a healthy 29-year-old woman The ADC and *D* values obtained with RT DWI were obviously higher in the left liver lobe (arrows) than those in the right. The differences of ADC and *D* values between the two lobes for RCT DWI were obviously lower than those for RT DWI. For RCT DWI, the *f* and D* maps of the liver showed relatively homogeneous, which indicated less variability of *f* and D* values compared with RT DWI.

The measurements of ADC, *D*, and *f* values varied significantly between the left and right liver lobe for both schemes (*P* < 0.001), except the *f* value for RT DWI (*P* = 0.499). As illustrated in Figures 2 and 4, the left liver lobe showed a tendency towards significantly higher values than the right liver lobe, which is more obvious for RT DWI. The left-to-right ratios of ADC and *D* values for RT DWI were 1.35–1.51, which were significantly higher than 1.09–1.16 for RCT DWI (Table 2, *P* ≤ 0.015). As for D* values, RT DWI resulted in higher values for the right liver lobe than those for the left liver lobe, which was opposite in RCT DWI, but for both schemes there was no significant difference of D* measurements between the two lobes (*P* ≥ 0.201).

**Table 2:** Left-to-right ratios (LRRs) of ADC and IVIM parameters measured at each DWI scheme and each session

Sequence	ADC	*D*	*f*	D*
**First Session**				
** RT**	1.51 ± 0.16	1.45 ± 0.18	1.18 ± 0.16	0.97 ± 0.45
** RCT**	1.14 ± 0.08	1.14 ± 0.09	1.06 ± 0.2	1.26 ± 0.58
*** * *P***	< 0.001	< 0.001	0.197	0.201
**Second Session**				
** RT**	1.42 ± 0.21	1.35 ± 0.24	1.19 ± 0.23	0.91 ± 0.50
** RCT**	1.09 ± 0.07	1.16 ± 0.11	0.89 ± 0.14	1.15 ± 0.42
*** * *P***	< 0.001	0.015	0.007	0.284

Data are mean ± standard deviation.

As far as the variability of ADC and IVIM parameters at the head-feet direction, CV of the values between the upper, middle, and lower section was evaluated. As summarized in Table 3, for both schemes, there is the lowest head-feet variability (6.5%–14.9%) for ADC and *D* values, the next for *f* value (13.74%–16.21%), and the largest variability for D* value (23.14%–28.77%). For RT DWI, the head-feet variability of ADC and *D* values among different sections was 12.47%–14.9%, which was significantly improved to 6.5%–7.65% (*P* ≤ 0.006) with RCT scheme. However, this improvement was not significant for *f* and D* values (*P* ≥ 0.334).

**Table 3:** Variability of ADC and IVIM parameters across the upper, middle and lower sections in the liver

Sequence	ADC	*D*	*f*	D*
**First Session**				
** RT (%)**	14.54 ± 4.96	12.47 ± 3.47	16.21 ± 6.65	27.92 ± 17.46
** RCT (%)**	7.65 ± 3.58	7.29 ±3.86	13.74 ± 7.62	24.43 ± 13.22
*** * *P***	0.006	0.003	0.334	0.536
**Second Session**				
** RT (%)**	14.9 ± 6.11	13.69 ± 4.40	14.15 ± 4.6	28.77 ± 11.88
** RCT (%)**	7.18 ± 3.70	6.50 ± 2.85	16.51 ± 7.22	23.14 ± 14.92
*** * *P***	0.006	0.002	0.453	0.375

Data are mean coefficient of variation ± standard deviation.

## DISCUSSION

DW MR imaging is susceptible to various kinds of motions, from microscopic diffusion of water molecules to macroscopic physiological motions, and the latter could lead to low SNRs, measurement error, enhanced variability, and decreased repeatability of ADC and IVIM parameters [7, 13]. For the liver, which located adjacent to the diaphragm, the effect of respiratory motion on DWI is even worse than for other organs, such as kidneys or the prostate. Furthermore, the left liver lobe was also susceptible to cardiac motion and stomach air, which may give rise to measurement error of ADC, *D*, and *f* values [13, 14]. Thus, it is extremely necessary to optimize physiological triggering scheme for the liver IVIM-DWI to improve image SNR and the precision, repeatability, and variability of ADC and IVIM parameters, especially to improve *f* and D* values, which have been shown poorer repeatability by many previous studies [8, 13]. Furthermore, the validation of optimization method should be systematically evaluated over different conditions. Therefore, in this study we applied additional cardiac triggering to RT DWI, which is a very common physiological triggering scheme for DWI acquisition in the clinical practice, and investigated whether the double triggering scheme would further optimize DWI protocol from the following aspects: SNR, the values of ADC and IVIM parameters, their repeatability, and variability across lobes and sections. To avoid the effects of other factors, like hardware and individuals, we performed this prospective study on the same volunteers with the same MR scanner. The DWI sequences shared the same parameters except the physiological triggering scheme.

On the basis of our results, we believe that RCT double triggering technique is an effective optimization scheme for liver IVIM-DWI, especially for the left liver lobe. Performing signal acquisitions during the diastolic phase of cardiac cycle, the heart-related signal loss has been significantly improved. Moreover, the over-estimation of ADC and IVIM parameters has also been significantly decreased, especially for the left liver lobe. One of the possible reasons for this phenomenon may be the reduction of measurement error of partial volume effect caused by the cardiac pulsation [14]. The cardiac motion may make the intrahepatic vessels, which usually show higher ADC and IVIM parameters than the liver tissue, overlapped on the liver parenchyma and lead to the over-estimation of quantitative values. In addition, the repeatability and variability of ADC and IVIM parameters were also significantly improved with different degree.

As our results showed, the advantage of adding cardiac triggering for RT DWI is not completely confined to the left liver lobe. Although the influence of additional cardiac triggering on the right liver lobe was not as obvious as the left liver lobe, the SNRs, over-estimation of parametric values, and repeatability of ADC, *D*, and *f* values had also been significantly improved with RCT DWI. Thierry et al. [12] found diffusion variability of the right liver lobe have also been decreased by using respiratory triggering with cardiac triggering (*P* = 0.001). Kwee et al. [15] performed liver DWI (*b* = 500 s/mm^2^) respectively at three orthogonal directions and found the effect of cardiac motion on the liver-to-background contrast also existed in the right liver lobe (left-right direction), which may explain the improvement in this study. However, another study which applied echocardiography triggering without RT for DWI failed to significantly optimize the values and test-retest repeatability of ADC and IVIM parameters for the right liver lobe compared with RT DWI, even for the test-retest repeatability of ADC and *D* values of the left liver lobe [13]. This may be because the uncontrolled respiratory motion offsets the optimization effect of cardiac triggering on the values and repeatability of ADC and IVIM parameters. Therefore, at this point, RCT double triggering is a better scheme to optimize liver DWI than single echocardiography triggering scheme.

Up to now, the RCT scheme for abdominal DWI has only been applied and evaluated in very limited studies. Thierry et al. [12] implemented respiratory triggering with cardiac respiratory triggering for liver DWI (*b* = 0–150–500 s/mm^2^) to investigate the repeatability of ADC values. They found the use of double triggering scheme reduced ADC differences between the left and right liver and significantly improved the variability and repeatability of diffusion quantification. Binser et al. [16] applied RCT technique for kidney DWI and obtained lower signal fluctuations and more reliable diffusion parameter measurements than RT DWI. So far, however, no published studies have investigated and evaluated the influence of RCT double triggering scheme on image SNRs and IVIM parameters for the liver DWI. The results of this study showed that compared with RT scheme, the use of RCT technique could not only significantly enhance SNRs of images, improve the over-estimation and repeatability of ADC, *D*, and *f* values, decrease the variability of ADC and *D* values, but also significantly improve the precision of D* values in the right liver lobe (*P* < 0.001) and its repeatability in the left lobe (*P* = 0.002). Although the potential of D* values has been shown for evaluating liver nonalcoholic fatty liver diseases and cirrhosis, meanwhile, its reliability is also doubted for its poor repeatability of measurements [9, 17]. Thus, further optimization technique seems more urgent for D* values before it is used as an imaging biomarker and RCT double triggering scheme is an available and reliable choice.

In both clinical and scientific research fields, the optimization of the triggering scheme is extremely significant, especially considering the widespread application of DWI and its quantitative parameters on detecting and characterizing diseases, and monitoring tumor response for radio- or chemotherapy [18–22]. The use of RCT double triggering scheme could decrease the motion of liver during scanning, which may reduce the partial volume of hepatic lesions from surrounding liver parenchyma, especially for the lesions in the left liver lobe [14]. The more precise and repeatable ADC and IVIM parameters may potentially be valuable for detection of more subtle structural changes of diseases at an early stage, which is always important for establishing diagnosis and therapy plan. In addition, both the longitudinal and horizontal comparison of quantitative parameters of hepatic diseases needs a more repeatable but less variability method for DWI acquisition. Therefore, we recommend that the RCT double triggering method is worth adopting for liver DWI in clinical context, if the patients are cooperative and acquisition time is allowing, 6–11 min in this study, especially for patients who need longitudinally evaluate and monitor the hepatic lesions with ADC or IVIM parameters.

There are limitations in this study. First, all the subjects in this study were young healthy volunteers, who were cooperative and had stable respiratory and heart rate. However, it is inevitably essential for accurately evaluating the potential benefit of a triggering technique to keep the feasibility of this optimization study on the subjects and to maintain good image quality of DW images. Just as we hypothesized, our study proved the improvement of SNRs and the repeatability and variability of quantitative parameters by using RCT scheme. However in patients, who might be less cooperative or suffering pain, the prolonged acquisition time of double triggering technique might be intolerable or offset its benefits to some degree. For patients with arrhythmia, the advantage of RCT for DWI may be decreased. Second, although we used nine *b* values for obtaining DWI images, the use of more *b* values, especially low *b* values (0–100 s/mm^2^), could lead to more accurate measurements of *f* and D* values. Third, in this preliminary study we did not include patients with any liver diseases. Therefore, whether RCT double triggering technique improves the evaluation of liver fibrosis and discrimination between various focal liver lesions still needs to be investigated by further studies. Fourthly, the reproducibility of ADC and IVIM parameters obtained with both methods in a longer period, or across different magnetic fields or vendors of MR system was not evaluated in this study.

In conclusion, we have investigated the influence of RCT double triggering scheme on IVIM-DWI and found that RCT DWI sequence resulted in a better combination of SNRs, precision, repeatability, and variability of ADC and IVIM parameters than RT DWI in the liver of volunteers. Hence, we believe that RCT double triggering technique is an effective method for optimizing liver DWI sequence and worth implementing for cooperative patients, especially whose quantitative parameters of DWI are crucial for making or changing the treatment plans.

## MATERIALS AND METHODS

### Participants

**Figure 5:**
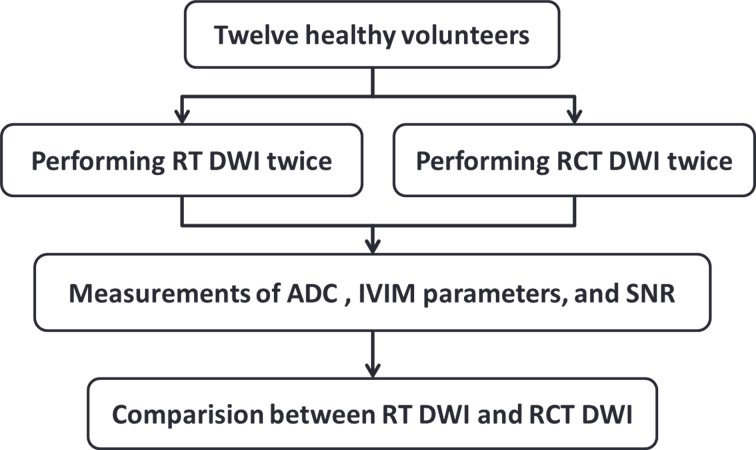
Flowchart of the study SNR = signal-to-noise ratio.

This prospective study was approved by the Ethics Committee of Xinhua Hospital Affiliated to Shanghai Jiao Tong University School of Medicine. Written informed consent was obtained from all the volunteers, and all the methods were performed in accordance with the approved guidelines and regulations. Twelve healthy adult volunteers (six women, six men; age: 26 ± 1.6 years) were enrolled in this study. All the volunteers had no prior history of diseases of the upper abdominal organs. To maintain a similar gastrointestinal state, the volunteers refrained from eating and drinking for 4–5 hours before imaging. The study procedure was as shown in the Figure 5.

### MR examinations

All the volunteers underwent DWI sequences at a 3.0-T MR imaging system (Ingenia, Philips Medical Systems, Best, the Netherlands). The maximal achievable gradient amplitude of this imager was 45 mT/m, and the slew rate was 200 T/m/s. A 32-channel phased-array torso coil was used to receive signals. The imaging protocol consisted of two DWI sequences respectively with RT and RCT scheme.

For RCT DWI, a peripheral pulse unit was used to detect the cardiac systole. According to previous studies [13, 16] and volunteer’s heart rate (mean, 63 bpm; range, 55–70 bpm), the trigger delay was set to 500 ms to maintain superior image quality for upper abdominal organs. Mean acquisition window was 458.4 ms (range, 357.1–590.9 ms). In RCT scheme, the expiration was first observed, and then signal acquisition is triggered at the defined delay time after the top of the pulse wave detected by the peripheral pulse unit.

The DWI sequences shared the following acquisition parameters: single-shot spin-echo echo-planar imaging sequence, trace nine *b* values (0, 20, 40, 70, 100, 150, 200, 500, 800 s/mm^2^), repetition time of three respiratory cycles (RT DWI) or of 3–4 respiratory cycles (RCT DWI), shortest time of echo (56 ms), parallel acceleration factor of four, echo train length 29, receiver bandwidth of 2640.5 Hz, field of view 292 mm × 353 mm × 178 mm, matrix 96 × 112, slice thickness of 7 mm, slice gap of 12 mm, 10 slices covering the upper abdomen, the number of signal averaged equals one, spectral presaturation with inversion recovery for fat suppression. Slices were kept locating in the same place between two schemes according to the anatomic structure such as the right branch of the hepatic portal vein. All the volunteers were out from the scanner for approximately 5 min after the first DWI acquisition and then received the second DWI series. The DWI parameters were identical for two sessions.

### Quantitative analysis and statistics

Quantitative image analysis was performed by a radiologist (J.N.L.) with 7 years of clinical experience in abdominal MR imaging. To explore the difference of ADC and IVIM parameters in the head-feet direction, five regions of interest (ROIs) were respectively drawn on the upper, middle, and lower section in the liver to measure ADC and IVIM parameters for each DWI sequence. Variability of quantitative parameters among the three sections was evaluated with CV. For each section, two of the five ROIs were placed at the left liver lobe, while the others were placed at the right liver lobe. Left-to-right ratio (LRR) was calculated for each parameter to evaluate the degree of difference of parametric values between the left and right liver lobe. During this process, ROIs with a standard size of 30 pixels were placed in the same locations on total four DWI acquisitions for each volunteer, avoiding large vessels and obvious artifacts. Then the signal intensity of each ROI was automatically detected for calculating ADC and IVIM parameters by using a DWI-Tool developed by Philips (IDL 6.3, ITT Visual Information Solutions, Boulder, CO, USA) [23] with a pixel-by-pixel basis. The total ADC was extracted from all nine *b* values and calculated based on the equation [7]:

$$\mathrm{SI}/{\mathrm{SI}}_{0}=e^{\left(-b\mathrm{ADC}\right)},$$

where SI represents the signal intensity with a given *b* value and SI_0_ is the signal intensity for *b* value of 0 s/mm^2^. The IVIM parameters were calculated with nonlinear least-squares curve fittings based on the Levenberg-Marquardt algorithm. The signal variation with the increase of *b* value could be written as [2]:

$$\mathrm{SI}/{\mathrm{SI}}_{0}=fe^{-bD*}+\left(1-f\right)e^{-bD}.$$

The repeatability—the variation under the same condition (obtained with the same MR scanner and DWI parameters) on the same subjects by a single observer in a short period of time (an interval of 5 min)—of ADC and IVIM parameters of each DWI scheme was assessed in terms of within-subject CV for each liver lobe.

In addition, SNRs were calculated for each *b* value by using a dedicated Philips post-processing workstation (IntelliSpace Portal). Mean signal intensity of each ROI with the same size and location as the ROI for ADC analysis was used for calculating SNR. Since the noise was prohibited by the use of parallel imaging, as recommended in the literature [24], we used the standard deviation (SD) of signal intensity of each ROI as an estimate of local noise.

Student *t* test was used for statistical comparison of the data sets between RT and RCT schemes and between the left and right liver lobes. Statistical analyses were performed with SPSS statistics version 23 software (IBM, Chicago, USA). Reported *P* values were two sided, and *P* < 0.05 was considered to be statistically significant.
